# Individualized lncRNA differential expression profile reveals heterogeneity of breast cancer

**DOI:** 10.1038/s41388-021-01883-6

**Published:** 2021-06-15

**Authors:** Zhangxiang Zhao, YingYing Guo, Yaoyao Liu, Lichun Sun, Bo Chen, Chengyu Wang, Tingting Chen, Yuquan Wang, Yawei Li, Qi Dong, Liqiang Ai, Ran Wang, Yunyan Gu, Xia Li

**Affiliations:** 1 Department of Systems Biology, College of Bioinformatics Science and Technology, Harbin Medical University, Harbin, China; 2 Department of Pharmacology (State-Province Key Laboratories of Biomedicine-Pharmaceutics of China Key Laboratory of Cardiovascular Research, Ministry of Education), College of Pharmacy, Harbin Medical University, Harbin, China; 3 Northern Translational Medicine Research and Cooperation, Heilongjiang Academy of Medical Sciences, Harbin Medical University, Harbin, China; 4 Department of Breast Medical Oncology, Harbin Medical University Cancer Hospital, Harbin, China; 5 Department of Physiology, Harbin Medical University, Harbin, China; 6 Department of Bioinformatics, College of Bioinformatics Science and Technology, Harbin Medical University, Harbin, China

**Keywords:** Tumour heterogeneity, Non-coding RNAs

## Abstract

Long non-coding RNAs (lncRNAs) play key regulatory roles in breast cancer. However, population-level differential expression analysis methods disregard the heterogeneous expression of lncRNAs in individual patients. Therefore, we individualized lncRNA expression profiles for breast invasive carcinoma (BRCA) using the method of LncRNA Individualization (*LncRIndiv*). After evaluating the robustness of *LncRIndiv*, we constructed an individualized differentially expressed lncRNA (IDElncRNA) profile for BRCA and investigated the subtype-specific IDElncRNAs. The breast cancer subtype-specific IDElncRNA showed frequent co-occurrence with alterations of protein-coding genes, including mutations, copy number variation and differential methylation. We performed hierarchical clustering to subdivide TNBC and revealed mesenchymal subtype and immune subtype for TNBC. The TNBC immune subtype showed a better prognosis than the TNBC mesenchymal subtype. LncRNA *PTOV1-AS1* was the top differentially expressed lncRNA in the mesenchymal subtype. And biological experiments validated that the upregulation of *PTOV1-AS1* could downregulate *TJP1* (ZO-1) and E-Cadherin, and upregulate Vimentin, which suggests *PTOV1-AS1* may promote epithelial-mesenchymal transition and lead to migration and invasion of TNBC cells. The mesenchymal subtype showed a higher fraction of M2 macrophages, whereas the immune subtype was more associated with CD4 + T cells. The immune subtype is characterized by genomic instability and upregulation of immune checkpoint genes, thereby suggesting a potential response to immunosuppressive drugs. Last, drug response analysis revealed lncRNA ENSG00000230082 (*PRRT3-AS1*) is a potential resistance biomarker for paclitaxel in BRCA treatment. Our analysis highlights that IDElncRNAs can characterize inter-tumor heterogeneity in BRCA and the new TNBC subtypes indicate novel insights into TNBC immunotherapy.

## Background

Long non-coding RNAs (lncRNAs) are involved in carcinogenesis through epigenetics, chromatin regulation, and transcriptional as well as post-transcriptional regulation [1, 2]. Moreover, lncRNAs are known as signatures for breast cancer classification or as potential prognostic biomarkers [3, 4]. Population-level lncRNA differential expression analysis has been used to identify differentially expressed lncRNAs in breast cancer. However, methods such as T-test are sensitive to technical factors, including different platforms and batch effects [5]. Despite inter-tumor heterogeneity, these methods disregard the differential expression of lncRNAs in a single patient. The fold-change (FC) method for pairwise comparison of disease and control samples is usually used to detect individual differentially expressed genes. However, the FC method lacks strict statistical control. Moreover, datasets with paired normal and cancer samples are rare in public databases. Recent methods to detect patient-specific differential expression based on relative gene expression have shown a great advantage; for example, the Rank Comparison (*RankComp*) method exhibits robustness to batch effects and data normalization [6]. Thus, *RankComp* can directly utilize data from different datasets to identify differentially expressed genes in individual samples. Our previous study proposed a LncRNA Individualization (*LncRIndiv*) method, which detects individualized differentially expressed lncRNAs (IDElncRNAs), to develop a prognostic signature for lung adenocarcinoma [7]. However, only a few studies have focused on analyzing IDElncRNAs to investigate the heterogeneity of breast cancer. Hence, identifying IDElncRNA may provide novel insights into the mechanism of known breast cancer subtypes and reveal new malignant breast cancer classification.

Breast cancer is a heterogeneous disease with different molecular subtypes that guide clinical treatment [8]. Prediction of microarray 50 (PAM50) has identified the following four stable subtypes: luminal A, luminal B, human epidermal growth factor receptor 2 (HER2)-enriched, and basal-like [9]. Clinicopathological subtypes were defined using immunohistochemistry markers: estrogen receptor (ER), progesterone receptor (PR), and HER2 [10–12]. The clinicopathological subtypes for breast cancer are classified as luminal A, luminal B, HER2+/HR+, HER2+/HR−, and triple-negative breast cancer (TNBC) in the Chinese society of clinical oncology (CSCO) guidelines. However, the role of lncRNAs in breast cancer subtypes remains unclear. Patients with the same breast cancer subtype respond differently to therapy and therefore have different clinical outcomes [13]. TNBC is the most aggressive breast cancer subtype and accounts for 10–20% of all breast cancer cases [14]. Under the same treatment strategy, diverse prognoses drive the need to explore potential TNBC subtypes with actionable targets [15–17]. Previous studies have revealed that TNBC is extremely heterogeneous and therefore this cancer type requires further classification. Thus, we aimed to explore the intrinsic differences in TNBC using the IDElncRNA profile.

In the present study, we constructed a breast cancer IDElncRNA profile using *LncRIndiv*. IDElncRNAs show differential DNA methylation or copy number variation (CNV). For breast cancer subtypes, IDElncRNAs reveal subtype-specific co-occurrence with alterations of protein-coding genes, indicating a co-operative regulatory role of lncRNAs in breast cancer progression. Some subtype-specific lncRNAs are associated with drug response. Moreover, clustering based on TNBC subtype-specific prognosis-related lncRNAs reveals immune and mesenchymal subtypes, where the immune subtype has been characterized by better prognosis, high genomic instability, and upregulation of immune checkpoint genes, thereby suggesting a potential response to immunosuppressive drugs.

## Materials and methods

### Data and preprocessing

Table S1 shows the statistics of samples and probes/genes in TCGA multi-omics data. See Supplementary Information for detail preprocesses.

### Evaluating the robustness of *LncRIndiv*

Using *LncRIndiv*, the quantitative lncRNA expression profile from the atlas of non-coding RNAs in cancer was transformed to an IDElncRNA profile, which defines lncRNA expression as upregulated, downregulated, or unaltered in each breast invasive carcinoma (BRCA) sample. From the lncRNA expression profile of 105 paired cancer-normal samples, we randomly selected 80% of overall paired samples (84 pairs) as the training set and the rest as the test set to perform a five-fold cross-validation test. The sample size of the normal samples was sufficient for stable lncRNA pair identification [6]. For each iteration, the *LncRIndiv* was applied to the training set to generate the IDElncRNAs’ reference criterion. To evaluate the performance of *LncRIndiv*, we validated IDElncRNAs in the test set. For example, if lncRNA-A was identified as upregulated/downregulated in the training set, we calculated its delta value (cancer-normal) in the test set. The average accuracy of lncRNA-A was defined as the number of positive/negative delta values divided by the total number of test sets. The average accuracy of both lncRNAs and samples was calculated.

### Identifying BRCA over-represented and subtype-specific lncRNAs

BRCA subtype information was available in The Cancer Genome Atlas (TCGA) following the classification standards: PAM50 and CSCO [18]. See Supplementary Information for details.

### Identifying prognosis-related lncRNAs and TNBC classification

See Supplementary Information for details.

### Pathway analysis of TNBC subtypes

See Supplementary Information for details.

### Characterization of the tumor immune microenvironment

The immunomodulator list and single nucleotide variants (SNV)-derived neoantigens were obtained from Vesteinn et al. [19]. The homologous recombination deficiency (HRD) score based on the loss of heterozygosity, telomeric allelic imbalance, and large-scale transitions were attained from the study of Knijnenburg et al. [20].

We extracted TCGA BRCA mRNA expression profile characterized by transcripts per million from gene expression omnibus (Accession number GSE62944) (https://www.ncbi.nlm.nih.gov/geo/) and performed CIBERSORT, TIMER, and xCELL methods to evaluate immune cell compositions [21–24]. LncRNA and its related immune pathways were attained from the ImmLnc database which calculated enrichment score (lncRES scores) for lncRNAs-pathways pairs (http://bio-bigdata.hrbmu.edu.cn/ImmLnc/jt-download.jsp) [25]. See Supplementary Information for details.

### Cell culture and transfection

See Supplementary Information for details.

### Wound healing assay

See Supplementary Information for details.

### Transwell assay

See Supplementary Information for details.

### Immunofluorescence

See Supplementary Information for details.

### RNA extraction and quantitative real-time PCR

See Supplementary Information for details.

### Protein extraction and western blot

See Supplementary Information for details.

### Validation of TNBC subtype in CCLE

Reverse phase protein array (RPPA) datasets of cell lines and pharmacologic profiles of 24 anticancer drugs across CCLE lines are available at https://data.broadinstitute.org/ccle/. The drug response was evaluated as activity area (ActArea) values. Cell lines were screened to obtain TNBC cell lines according to the receptor status reported in a previous review [26]. See Supplementary Information for details.

### Identifying BRCA drug response-related IDElncRNA

See Supplementary Information for details.

### Statistical analysis

See Supplementary Information for details.

## Results

### Analytic pipeline of IDElncRNA profile for BRCA

We employed the *LncRIndiv* method to construct an IDElncRNA profile for BRCA using the lncRNA expression profile from TCGA (Fig. S1A). We then identified BRCA subtype over-represented IDElncRNAs and BRCA subtype-specific IDElncRNAs (Fig. S1B). We also investigated the co-occurrence between differential expression of lncRNAs and alterations of protein-coding genes, including mutations, CNV, and differential methylation in BRCA (Fig. S1C). Further, we performed clustering analysis to reveal novel TNBC subtypes with remarkably different prognoses and tumor-infiltrating immune cells (Fig. S1D). The top candidate lncRNA was validated by biological experiments. Finally, we assessed potential drug response-related lncRNAs for breast cancer using IDElncRNA (Fig. S1E).

### Evaluating the accuracy and reliability of IDElncRNAs

**Fig. 1:**
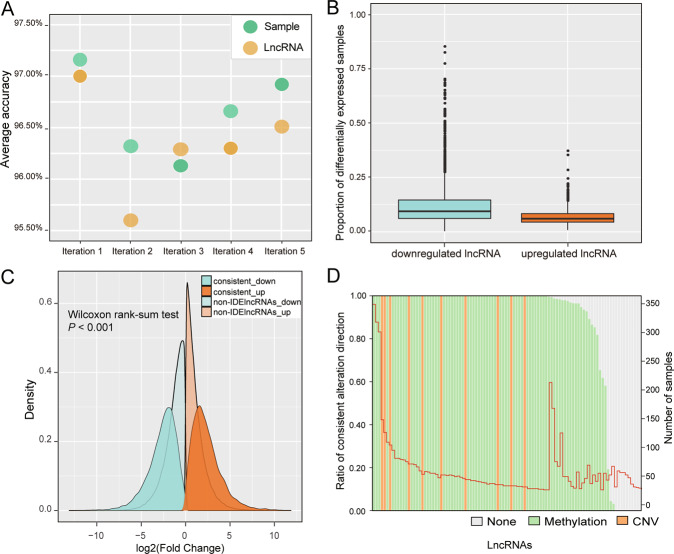
Performance evaluation of *LncRIndiv*. **A** Average accuracy of five-fold cross-validation. **B** IDElncRNAs profile statistics for breast cancer. Horizontal axis represents groups of upregulated lncRNA and downregulated lncRNA, and the vertical axis indicates the proportion of samples with differentially expressed lncRNA in all breast cancer samples. **C** FC distribution of IDElncRNAs with consistent differential expression direction and non-IDElncRNAs. Red and blue colors indicate the direction of fold change. Dark and light colors represent IDElncRNAs and non-IDElncRNAs, respectively. **D** The sample statistics of IDElncRNAs with consistent alteration direction between IDElncRNAs and CNV or DNA methylation. The left ordinate indicates the ratio of consistency and the right ordinate means the number of samples with IDElncRNAs, which is marked with a red line. LncRIndiv lncRNA individualization, lncRNA long non-coding RNAs, IDElncRNAs individualized differentially expressed long non-coding RNAs, CNV copy number variation, FC fold-change.

Overall, 3,458 lncRNAs were included in the IDElncRNA profile, which was determined using 10,047 lncRNA pairs (Table S2). The mean accuracy of the five-fold validation test was above 95% at both the sample and lncRNA levels (Fig. 1A). In the IDElncRNA profile, 1,909 lncRNAs were downregulated and 1,549 lncRNAs were upregulated. On average, lncRNAs were differentially expressed in 9.8% BRCA samples. The downregulated and upregulated lncRNAs accounted for 12.2% and 6.8% of BRCA samples, respectively, indicating that IDElncRNAs tend to be inhibited in BRCA (Fig. 1B).

To compare differential expression from FC and *LncRIndiv*, we divided 3,458 lncRNAs into two groups: IDElncRNAs with consistent FC direction and non-IDElncRNAs. Here, in 105 paired cancer-normal BRCA samples, FC distribution of upregulated IDElncRNAs with consistent FC direction was greater than that of non-IDElncRNAs (median log2 [FC] = 2.11 and 0.88, *P* < 0.001, Fig. 1C). Moreover, downregulated IDElncRNAs also displayed the same tendency (median log2 [FC] = − 2.76 and −1.09, *P* < 0.001, Fig. 1C). These results suggested that IDElncRNAs tend to have a greater magnitude of changes and be more likely differently expressed than that of non-IDElncRNAs.

Differential expression of lncRNAs may be the consequence of genomic or epigenetic alterations [27, 28]. Therefore, we further investigated CNV and DNA methylation of lncRNAs. Among 3,458 lncRNAs derived from *LncRIndiv*, 48 lncRNAs appeared in the CNV region and 2,542 lncRNAs had corresponding probes in the promoter region of the methylation microarray. Hence, we assumed that amplification or hypomethylation induces upregulation of lncRNAs, whereas hypermethylation or deletion leads to downregulation of lncRNAs. For the top 100 most frequent differentially expressed lncRNAs, we estimated the consistency for each lncRNA, where consistency means the lncRNAs upregulated in one breast cancer sample also showed amplifications or hypomethylation, and vice versa. Sixty-six lncRNAs’ differential expression was 100% consistent and 24 lncRNAs were partially consistent with CNV or DNA methylation, whereas 10 lncRNAs showed no consistency with CNV or DNA methylation (Fig. 1D). LncRNAs’ differential expression is related to either abnormal DNA methylation or CNV (Fig. 1D). Moreover, DNA methylation may be the major cause of lncRNAs’ differential expression in individual BRCA samples. Hence, the consistency between lncRNAs’ differential expression and CNV or differential methylation indicated the reliability of *LncRIndiv*.

### BRCA subtypes with the same receptors have common IDElncRNAs

**Fig. 2:**
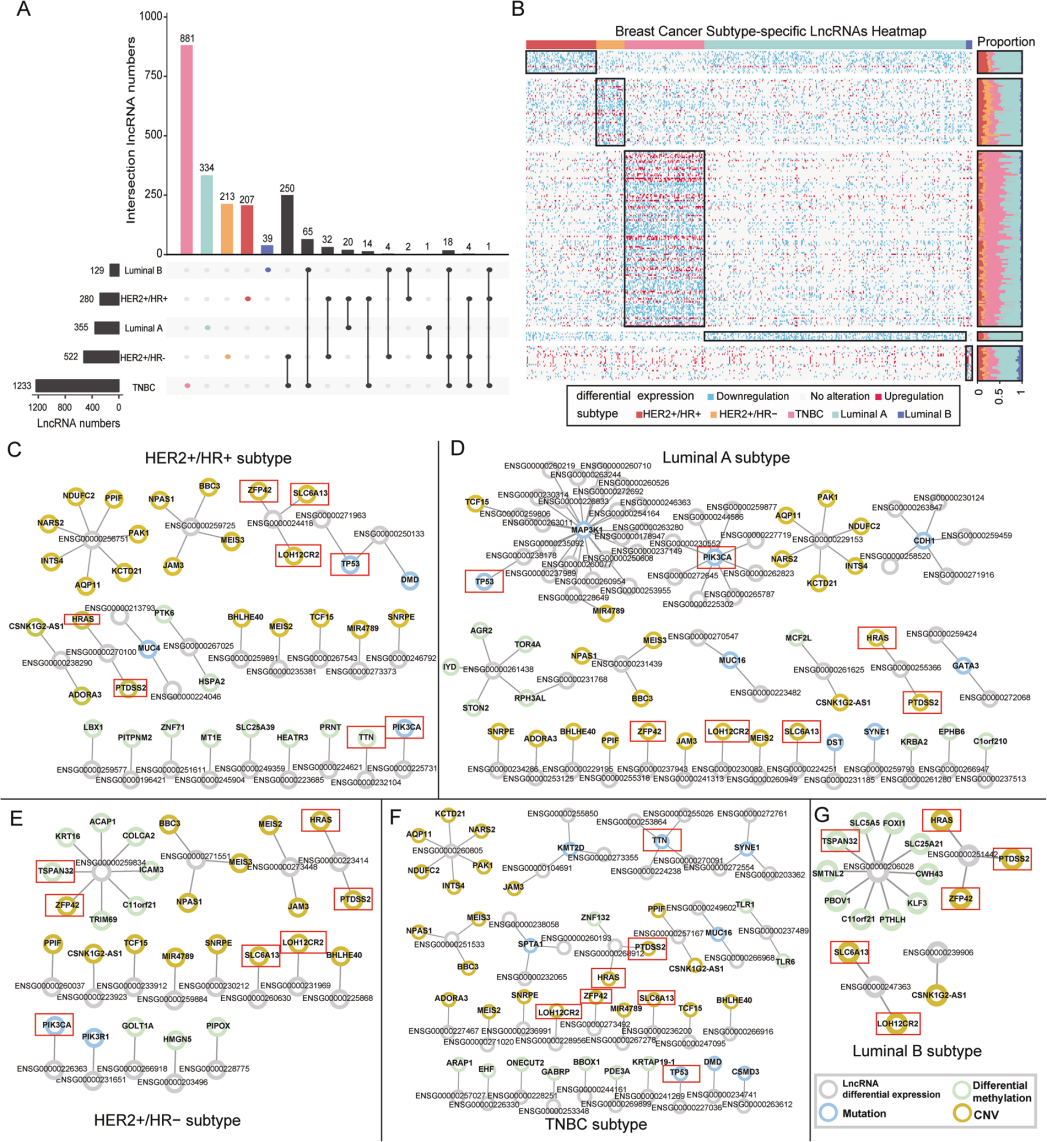
BRCA subtype-specific lncRNAs and co-occurrence network. **A** UpSet diagram shows the subtype-specific lncRNAs and intersections of over-represented lncRNAs among breast cancer subtypes. The black horizontal bar graphs indicate the number of over-represented lncRNAs of each subtype. Black circles show the intersections of subtype over-represented lncRNAs, and the black vertical bar graphs show the number of overlapped subtype over-represented lncRNAs. Colored bars represent the amount of subtype-specific lncRNAs. **B** Heatmap of breast cancer subtype-specific lncRNAs. The row and column represent lncRNAs and samples, respectively. The black rectangle indicates each subtype of breast cancer and corresponding subtype-specific lncRNAs. The right bar displays the proportion of BRCA samples in which the subtype-specific lncRNAs are differentially expressed. For clarity, the lncRNAs that are differentially expressed in >5% of samples within each subtype have been presented. Co-occurrence network in (**C**) HER2+/HR+; (**D**) Luminal A; (**E**) HER2+/HR−; (**F**) TNBC; (**G**) Luminal B. Nodes represent lncRNAs or protein-coding genes, and edges indicate co-occurring alterations between lncRNAs and protein-coding genes. Node color represents the type of alteration of protein-coding genes: yellow nodes depict genes with CNV; green nodes indicate genes with differential methylation; blue nodes represent genes with mutation and gray nodes represent subtype-specific IDElncRNAs. Red rectangles represent cancer-related genes, which were frequently co-altered with lncRNAs in multiple breast cancer subtypes. lncRNA long non-coding RNAs, IDElncRNAs individualized differentially expressed long non-coding RNAs, HER2 +  human epidermal growth factor receptor 2, TNBC triple-negative breast cancer, CNV copy number variation.

Following the CSCO subtype classification, the TNBC subtype was the most aggressive and had the most over-represented IDElncRNAs, followed by the HER2 + /HR − subtype and luminal A subtype (Fig. 2A). We found 250 over-represented lncRNAs in common between the TNBC and the HER2 + /HR − subtype, within which they share negative ER and PR. TNBC subtypes shared 65 over-represented lncRNAs with luminal B subtypes, despite the minimal number of over-represented lncRNAs in the luminal B subtype. Thus, the luminal B and TNBC subtypes may have similar mechanisms at the lncRNA level. Luminal A and HER2 + /HR − subtypes, sharing no hormone receptor and HER2 status, had only one common lncRNA (Fig. 2A). Subtype-specific IDElncRNAs with high frequency are shown in Fig. 2B. Besides, subtype-specific lncRNAs of BRCA PAM50 subtypes are listed in Fig. S2. Thus, the overlaps of over-represented lncRNAs among different BRCA subtypes suggest that subtypes with the same hormone receptor status tend to have common IDElncRNAs.

### Subtype-specific IDElncRNAs cooperate with other molecular alterations

TNBC had the greatest number of subtype-specific lncRNAs among all BRCA subtypes (Fig. 2A). LncRNAs regulate DNA repair and methylation by binding to proteins and DNA [29, 30]. Thus, IDElncRNAs may cooperatively alter the genetic and epigenetic modifications of protein-coding genes. Differential expression of subtype-specific IDElncRNAs showed significant co-occurrence with CNV, somatic mutation, or differential methylation of protein-coding genes in each subtype (Fig. 2C–G). Notably, CNV of *HRAS*, *PTDSS2*, *ZFP42*, *LOH12CR1*, and *SLC6A13* showed significant co-occurrence with IDElncRNAs in all subtypes (*P* < 0.05). Somatic mutations in *TP53* and *PIK3CA* showed co-occurrence with IDElncRNAs in four subtypes, except luminal B (Fig. 2C–F). As for differential methylation, only tumor suppressor gene *TSPAN32* co-occurred with IDElncRNAs in HER2 + /HR − subtype and luminal B subtype (Figs. 2E, G). Moreover, methylation and mutation of the *TTN* gene showed co-occurrence with IDElncRNAs in the HER2+/HR+ subtype and TNBC subtype, respectively.

Hub subtype-specific lncRNAs, such as *AL157394.1* (ENSG00000261438), *RP11-284N8.3* (ENSG00000259834), and *Z99774.1* (ENSG00000206028), may participate in the progression of BRCA subtype by cooperation with other alterations of coding genes. In the luminal A subtype, lncRNA *AL157394*.1 showed co-occurrence with differential methylation of some genes (*AGR2*, *STON2*, and *RPH3AL*), which are related to cell trafficking function (*P* = 1.01E10-7 for *AGR2*, *P* = 3.97E10-7 for *STON2*, and *P* = 5.13E10-9 for *RPH3AL*, Fig. 2D). *RP11-284N8.3* plays an essential role in T-cell activation and co-occurred with differential methylation of immune system-related genes (*P* = 1.1E10-6 for *ICAM3* and *P* = 3.09E10-5 for *TRIM69*, Fig. 2E). In addition, colon cancer-related genes were also involved in the co-occurrence with *RP11-284N8.3* (*P* = 1.1E10-6 for *ACAP1* and *P* = 3.09E10-5 for *COLCA2*, Fig. 2E). In the luminal B subtype, *SLC5A5* and *SLC25A21* of solute carrier family as well as *FOXI1* and *KLF3* of DNA-binding family co-occurred with *Z99774.1* (*P* = 0.029 for *SLC5A5*, *SLC25A21*, *FOXI1*, and *KLF3*, Fig. 2G). The above results suggest that the subtype-specific lncRNAs could be involved in the BRCA subtypes by the coordinated alteration with mutations, CNV, or differential methylation of coding genes.

### IDElncRNA profile reveals novel TNBC subtypes

TNBC, the most malignant BRCA subtype, has the greatest number of IDElncRNAs among BRCA subtypes (Fig. 2A). Based on the 27 TNBC survival-related lncRNAs (Table S3), 90 TNBC samples were divided into two classes, consisting of 67 samples (Class 1) and 23 samples (Class 2).

**Fig. 3:**
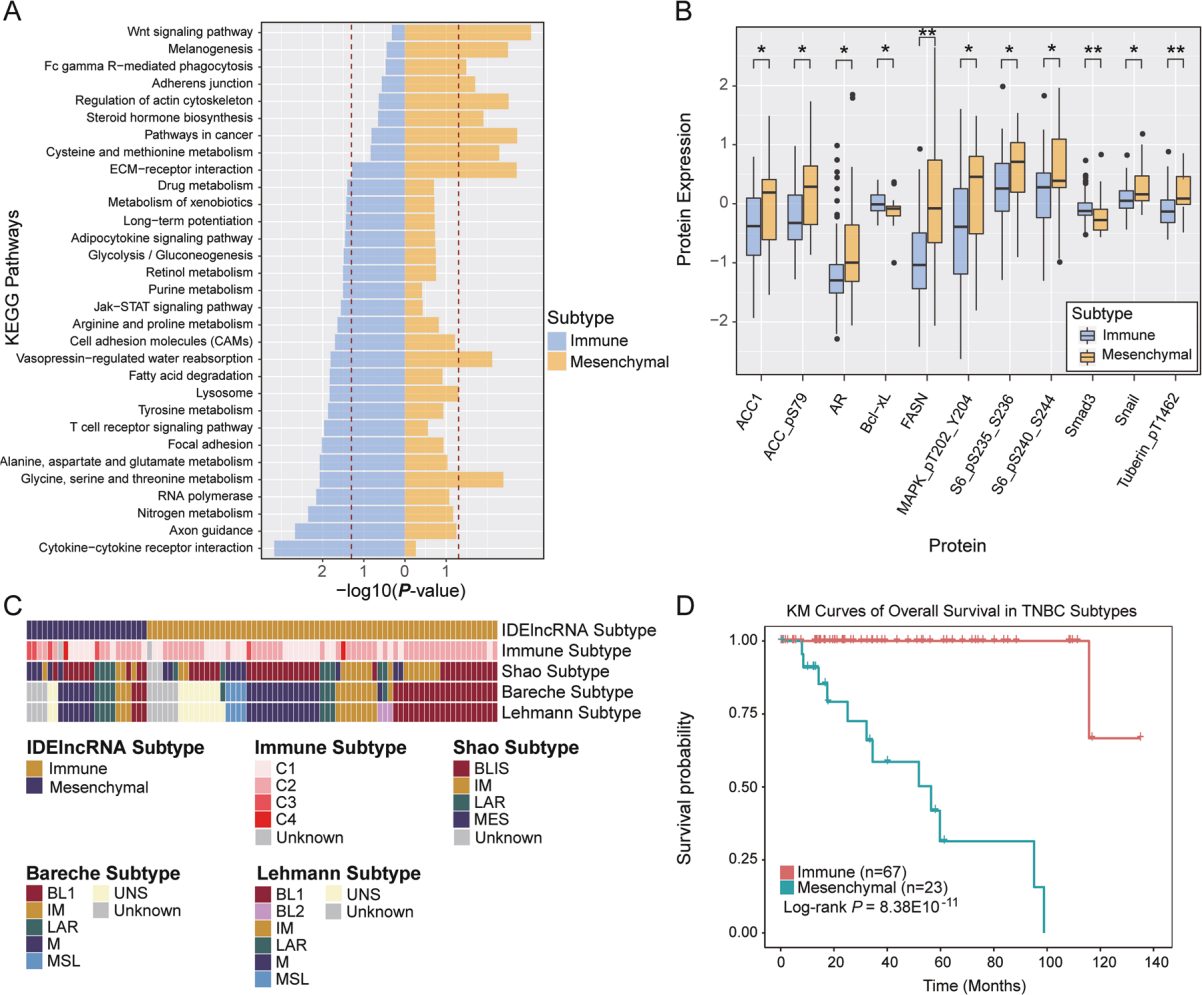
Characterization of TNBC subtypes. **A** KEGG pathway enrichment analysis of TNBC subtype-specific protein-coding genes. The dashed red vertical line corresponds to *P* = 0.05. **B** Significant differential expression of proteins between immune subtype and mesenchymal subtype. The data were analyzed using the Wilcoxon rank-sum test, and *P* < 0.05 was considered statistically significant. **C** Comparative analysis of TNBC subtypes derived from other studies. **D** The survival analysis of immune subtype and mesenchymal subtype. *P < 0.05, **P < 0.01, ***P < 0.001. TNBC triple-negative breast cancer, KEGG Kyoto encyclopedia of genes and genomes.

To characterize the two classes, we identified Class 1 and Class 2 specific protein-coding genes as well as related pathways. Class 1 was enriched with differential expression of genes involved in cytokine–cytokine receptor interaction, JAK-STAT signaling pathway, and T-cell receptor signaling (*P* < 0.05, Fig. 3A), suggesting that Class 1 tends to deregulate the immune system. Hence, we defined Class 1 as the immune subtype. For Class 2, differentially expressed genes were enriched in the Wnt signaling pathway, adherens junction, and extracellular matrix-receptor interaction pathways (*P* < 0.05, Fig. 3A). Hence, Class 2 was defined as the mesenchymal subtype.

As two TNBC subtypes have different transcriptomic characteristics, we further investigated the differences in protein expression. Proteins MAPK was notably expressed in the mesenchymal subtype (*P* = 0.03, Fig. 3B). SNAIL protein, a prominent epithelial-mesenchymal transition (EMT) inducer, showed higher expression in the mesenchymal subtype than in the immune subtype (*P* = 0.023, Fig. 3B). In addition, SMAD3 protein in the TGF-beta signaling pathway and Bcl-xL protein in the JAK-STAT signaling pathway were upregulated in the immune subtype (*P* = 0.0081 for SMAD3 and *P* = 0.027 for Bcl-xL, Fig. 3B). Expression of other proteins (ACC, FASN, S6, and tuberin) was evaluated in the mesenchymal subtype, which indicated the metabolic difference between the two subtypes of TNBC. Expressions of ACACA, AR, FASN and SNAI1 at mRNA levels are significantly upregulated in mesenchymal subtype compared with immune subtype of TNBC (Fig. S3).

Moreover, we compared our IDElncRNA subtype with previously published subtypes. Our subtypes were significantly associated with the immune subtype from Thorsson et al. [19]. (*P* = 0.021, Fig. 3C). Most TNBC tumors were classified as the C2 immune subtype by Thorsson et al. In our study, 63.6% immune subtype overlapped with C2 and 45.5% mesenchymal subtype was C2. The mesenchymal subtype tended to overlap with C3 (*P* = 0.013, Fisher’s exact test). In addition, our subtypes showed no significant association with Shao, Bareche, and Lehmann subtypes. Moreover, we found that TNBC patients with mesenchymal subtype had a poorer prognosis than TNBC patients with immune subtypes (*P* = 8.38E10-11, Log-rank test, Fig. 3D).

### TNBC subtypes are characterized by multi-omics data alterations

**Fig. 4:**
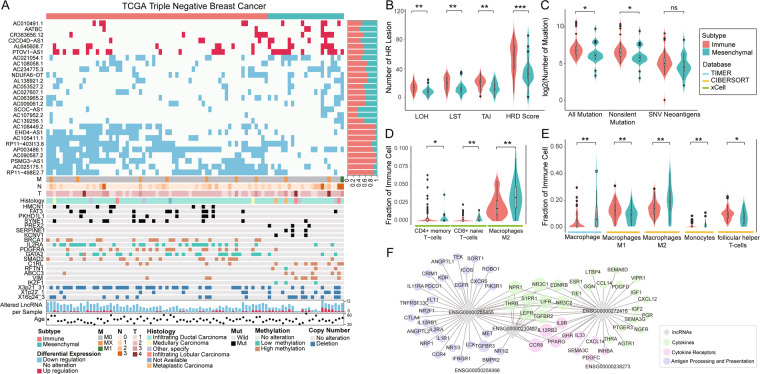
Characterization of the genome instability and immune cell infiltration between TNBC subtypes. **A** Multi-omics characterization of TNBC subtypes. Rows from top to bottom represent the subtypes of TNBC samples, individualized lncRNAs expression profile, lncRNA alteration ratio, stages, histology, gene mutations, DNA methylations, CNV regions, numbers of lncRNA alteration, and ages. The distribution of (**B**) HRD score and (**C**) TMB for patients in two subtypes. *P* values were calculated using Wilcoxon’s rank-sum tests. Tumor immune infiltration estimation from (**D**) xCell, (**E**) TIMER, and CIBERSORT. **F** Co-expression network of lncRNA-pathway pairs from ImmLnc. *P < 0.05, **P < 0.01, ***P < 0.001. TNBC triple-negative breast cancer, lncRNA long non-coding RNAs, CNV copy number variation, TMB tumor mutational burden, HRD homologous recombination deficiency.

We integrated multi-omics data to identify characteristic alterations for two TNBC subtypes at the genomic and epigenetic levels. At the epigenetic level, the immune subtype showed higher frequencies of differential methylation of *BRCA1*, *IL2RA*, *GATA2*, and *SMAD2* than those in the mesenchymal subtype (Fig. 4A). The hypermethylated *BRCA1* supports BRCAness phenotype and causes HRD. TNBC patients with the immune subtype had significantly higher HRD scores than patients with the mesenchymal subtype (*P* = 0.00083, Fig. 4B). Interestingly, in the immune subtype, we observed a higher frequency of the hypomethylated *IL2RA*, which encodes the CD25 marker of regulatory T-cells, than that in the mesenchymal subtype*. PDGFRA*, a cell surface tyrosine kinase receptor secreted by macrophages, appeared at a higher frequency of hypermethylation in the immune subtype than in the mesenchymal subtype (Fig. 4A).

Contrastingly, patients with the mesenchymal subtype tended to have higher frequencies of hypermethylation in *VIM* and mutations in *LAMA1* (Fig. 4A). No CNV region was considerably over-represented in any TNBC subtype. Clinical factors, including stage evaluating tumor size, lymph node metastasis, distant metastasis, age, and histology, showed no significant differences between immune subtype and mesenchymal subtype.

### TNBC subtypes show distinct immune microenvironment

To characterize the tumor immune microenvironment between TNBC subtypes, we compared the expression of immunomodulators including PD-1 and PD-L1, Tumor mutational burden (TMB), and HRD score between the two subtypes. The results showed that both *PDCD1* and *CD274*, which encode proteins PD-1 and PD-L1, respectively, showed increased upregulation frequency in the immune subtype (*CD274*: 14/52 versus 2/20, *P* = 0.34, Fisher’s exact test; *PDCD1*: 42/24 versus 7/15, *P* = 0.013, Fisher’s exact test). Moreover, *CTLA4*, acting as a major negative regulator of T-cell responses, also exhibited increased upregulation frequency in the immune subtype *(P* = 0.013, Fisher’s exact test).

TMB is a genomic biomarker that predicts favorable responses to immune checkpoint inhibitors. Patients in the immune subtype had significantly more mutations and non-silent mutations than that of the mesenchymal subtype (*P* = 0.017 for all mutations and 0.013 for non-silent mutations, Fig. 4C), whereas SNV neoantigen load difference was marginally significant (*P* = 0.066, Fig. 4C). These results suggest that genomic instability in the immune subtype may induce neoantigenic immune targets, and patients with the immune subtype express increased expression of immune system inhibiting genes to achieve immune evasion.

We further assessed the fractions of tumor-infiltrating immune cells in our two TNBC subtypes. Based on xCell, TIMER, and CIBERSORT, we found that macrophage infiltration was consistently higher in the mesenchymal subtype than in the immune subtype (Fig. 4D–E). Specifically, results from both CIBERSORT and xCell supported a higher fraction of infiltrating macrophage M2 in the mesenchymal subtype than in the immune subtype (*P* = 0.006 for CIBERSORT and *P* = 0.008 for xCell, Fig. 4D–E). Various T-cells, including CD4 + memory T-cells and follicular helper T-cells, showed higher infiltration of immune cells in the immune subtype than in the mesenchymal subtype (Fig. 4D).

### IDElncRNAs regulate immune pathway in TNBC

To gain insight into the function of 27 lncRNAs in immune regulation, we examined the lncRNA-pathway pairs that were identified by the ImmLnc database and constructed a co-expression regulatory network with immune genes [25]. Twelve of 27 lncRNAs were co-expressed with immune pathway-related genes, such as TNF family member receptors, interleukin receptors, antimicrobials, cytokine receptors, cytokines, and antigen processing and presentation. However, only genes expressing cytokine receptors, cytokines, and involved in antigen processing and presentation pathways displayed significant co-expression with IDElncRNA in all TNBC samples. Immunomodulators, including *PDCD1* and *CTLA4*, were co-expressed with lncRNA ENSG00000255455 (*RP11-890B15.3*), indicating that ENSG00000255455 is a key regulator of immune evasion in the immune subtype (adjusted *P* < 0.1, Fig. 4F).

### LncRNA *PTOV1-AS1* regulates the EMT process in MDA-MB-231 cells

**Fig. 5:**
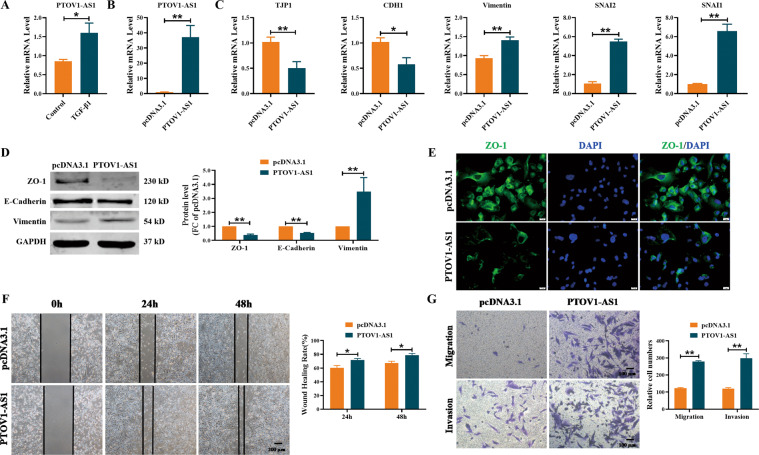
Overexpression of lncRNA *PTOV1-AS1* promotes EMT process in MDA-MB-231 cells. **A** qRT-PCR analysis of the expression of lncRNA *PTOV1-AS1* in MDA-MB-231 cells. *n* = 4. **B**–**C** The expression of *PTOV1-AS1* and EMT-related genes in MDA-MB-231 cells with transfection of *PTOV1-AS1* overexpression plasmid or pcDNA3.1. *n* = 5. **D** Western blot analysis of epithelial markers ZO-1 and E-Cadherin and mesenchymal marker Vimentin in MDA-MB-231 cells with transfection of *PTOV1-AS1* overexpression plasmid. *n* = 4. **E** Epithelial marker ZO-1 expression was determined by immunofluorescence in MDA-MB-231 cells. ZO-1 is stained green and the nucleus is stained blue. *n* = 4. Scale bar, 20 μm. **F** Wound healing assay showed overexpression of *PTOV1-AS1* promoted wound closure of MDA-MB-231 cells. *n* = 6. Scale bar, 200 μm. **G** The transwell assay was used to detect the effect of *PTOV1-AS1* overexpression on cell migration and invasion ability. *n* = 6. Scale bar, 100 μm. *P < 0.05; **P < 0.01. lncRNA long non-coding RNAs, EMT epithelial mesenchymal transition, qRT-PCR real-time quantitative reverse transcription PCR.

LncRNA *PTOV1-AS1* had the highest frequency of differential expression in the mesenchymal subtype. Both *PTOV1-AS1* and lncRNA *AATBC* were increased in the MDA-MB-231 cells treated with TGF-β1 (Figs. 5A and S4). To further explore the functional effect of *PTOV1-AS1* on the EMT process, we transfected *PTOV1-AS1* overexpression plasmid into MDA-MB-231 cells (Fig. 5B). We found that forced expression of *PTOV1-AS1* resulted in the downregulation of *TJP1* (ZO-1) and *CDH1* (E-Cadherin) and upregulation of Vimentin and *SNAI1/2* at mRNA levels (Fig. 5C). Meanwhile, the overexpression of *PTOV1-AS1* decreased the expression of ZO-1 and E-Cadherin and increased the expression of Vimentin at protein levels (Fig. 5D). Moreover, immunofluorescence assays further confirmed that overexpression of *PTOV1-AS1* could significantly reduce the staining intensity of ZO-1 in MDA-MB-231 cells (Fig. 5E). As illustrated in Fig. 5F–G, enhanced expression of *PTOV1-AS1* promoted wound healing ability and increased the migration and invasion in MDA-MB-231 cells. The above results suggest that the overexpression of *PTOV1-AS1* can trigger EMT process, and promote migration and invasion in MDA-MB-231 cells.

**Fig. 6:**
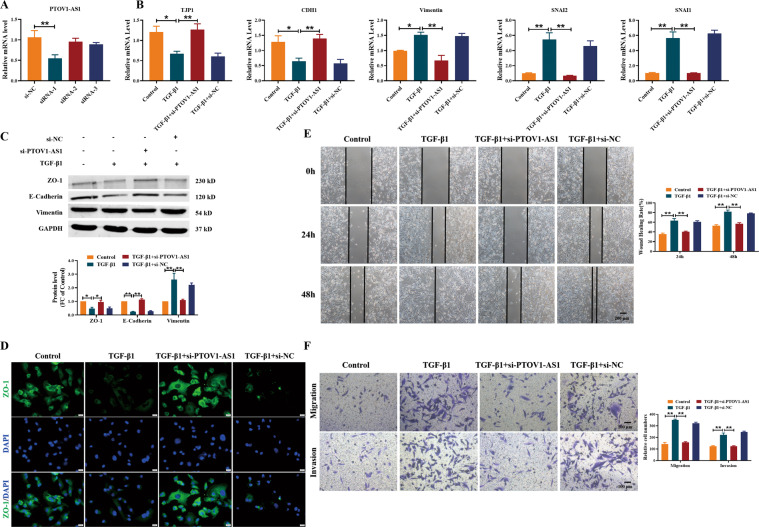
Silencing of lncRNA *PTOV1-AS1* impedes TGF-β1-induced EMT process in MDA-MB-231 cells. **A**–**B** qRT-PCR showed the inhibitory effect of si-PTOV1-AS1 on EMT in MDA-MB-231 cells treated with TGF-β1. *n* = 4. **C** The inhibitory effects of si-PTOV1-AS1 on TGF-β1-induced EMT were detected by Western blot. *n* = 4. **D** Immunofluorescence staining of ZO-1 revealed the inhibitory effect of si-PTOV1-AS1 on the EMT process in MDA-MB-231 cells. *n* = 4. Scale bar, 20 μm. Wound healing assay (**E**) and Transwell assay (**F**) showed that silencing *PTOV1-AS1* attenuated TGF-β1-induced cell migration and invasion. *n* = 6. Scale bar, 200 μm in (**E**) and 100 μm in (**F**). *P < 0.05; **P < 0.01. lncRNA long non-coding RNAs, EMT epithelial mesenchymal transition, qRT-PCR real-time quantitative reverse transcription PCR.

Then, we constructed siRNA against *PTOV1-AS1* (si-PTOV1-AS1) to further explore the function of *PTOV1-AS1* knockdown on the wound closure, migration, and invasion in MDA-MB-231 cells. As illustrated in Fig. 6A–C, silencing of *PTOV1-AS1* resulted in the upregulation of *TJP1* and *CDH1* and the downregulation of Vimentin and *SNAI1/2* both at mRNA and protein levels. Meanwhile, TGF-β1 inhibited the expression of ZO-1, whereas that was reversed by si-PTOV1-AS1 (Fig. 6D). Moreover, knockdown of *PTOV1-AS1* attenuated the TGF-β1-induced wound closure, migration, and invasion in MDA-MB-231 cells (Fig. 6E–F). Thus, these results showed that silencing *PTOV1-AS1* can alleviate TGF-β1-induced EMT and migration in MDA-MB-231 cells.

### Evaluating the robustness of TNBC classification in cell lines

**Fig. 7:**
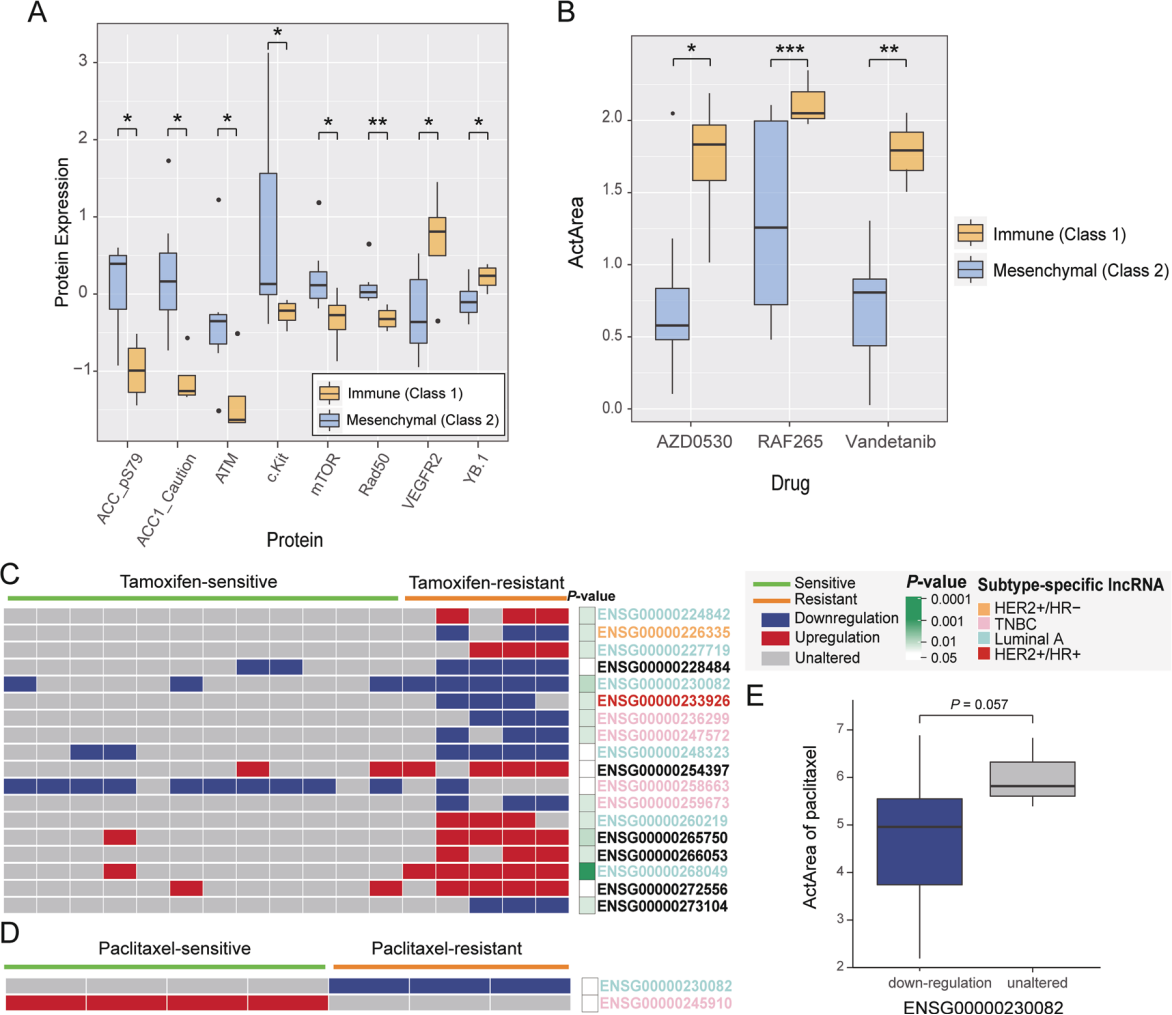
TNBC cell line subtype and drug response analysis. **A** Significant differential expression of proteins between TNBC immune subtype and mesenchymal subtype cell lines was tested by Wilcoxon rank-sum test. *P* < 0.05 was considered statistically significant. **B** Significant differential drug response of three drugs between TNBC immune subtype and mesenchymal subtype were tested by Welch’s *t* test. *P* < 0.05 was considered statistically significant. **C**–**D** Individual differential expression profile of drug response-related lncRNAs for (**C**) tamoxifen and (**D**) paclitaxel. Columns represent patients treated with drugs, and *P* values were derived from Fisher’s exact test. Rows represent drug response-related lncRNAs colored by the corresponding subtype. Red, blue, and gray rectangles indicate upregulated, downregulated, and unaltered lncRNAs, respectively. **E** ActArea values of paclitaxel were analyzed using Welch’s *t* test. Statistical significance is indicated by ****P < 0.0001, ***P < 0.001, **P < 0.01, *P < 0.05, and non-significant difference (ns). TNBC triple-negative breast cancer, lncRNA long non-coding RNAs, IDElncRNAs individualized differentially expressed long non-coding RNAs, ActArea activity area.

To examine the robustness of the newly discovered subtypes from TCGA TNBC samples, a hierarchical clustering analysis was conducted using 27 prognostic IDElncRNAs in CCLE TNBC cell lines. According to breast cancer classification in a previous review [26], 13 TNBC cell lines were classified into two classes (four in Class 1 and nine in Class 2). We found that fatty acid synthesis-related proteins such as ACC1 and phospho-ACC (Ser79, ACC_pS79) displayed significantly increased expression in Class 2, which was also notably expressed in the TNBC mesenchymal subtype (*P* = 0.024 for ACC_pS79 and *P* = 0.012 for ACC1, Fig. 7A). Moreover, the DNA repair genes, *ATM* and *RAD50*, were downregulated in Class 1 cell lines, indicating the genomic instability in Class 1 cell lines. These results imply that Class 1 cell lines correspond to the immune subtype and Class 2 cell lines correspond to the mesenchymal subtype, which supports IDElncRNA-based classification in tissue samples. We then investigated the anticancer drug response of 24 drugs. The drug responses of AZD0530, RAF265, and Vandetanib displayed lower ActArea values in mesenchymal cell lines than in immune cell lines (*P* = 0.02 for AZD0530, 0.0001 for RAF265, and 0.007 for Vandetanib, Fig. 7B). Specifically, mesenchymal cell lines showed downregulation of VEGFR2 protein (*P* = 0.042, Fig. 7A), which is targeted by Vandetanib.

### Identifying drug response-related IDElncRNAs for BRCA

LncRNAs’ differential expression can be used as potential drug response biomarkers [31]. In TCGA BRCA samples, 18 IDElncRNAs (17 drug resistance-related and 1 sensitivity-related lncRNAs) and 2 IDElncRNAs (1 drug resistance-related and 1 sensitivity-related lncRNAs) were found to be associated with drug response to tamoxifen and paclitaxel, respectively (Fig. 7C, D). For instance, BRCA patients with differential expression of ENSG00000245910 were sensitive to paclitaxel (*P* = 0.029, Fisher’s exact test), and BRCA patients with differential expression of ENSG00000258663 were sensitive to tamoxifen (*P* = 0.028, Fisher’s exact test). As tamoxifen was used to treat ER- breast cancer, we found that 7 tamoxifen response-related lncRNAs were either luminal A or HER2- subtype-specific lncRNAs (Fig. 7C). CCLE data have investigated anticancer drug responses, including paclitaxel, in 51 breast cancer cell lines. Cell lines with downregulated ENSG00000230082 (*PRRT3-AS1*) showed lower ActArea values after paclitaxel treatment than in cell lines with unaltered ENSG00000230082 (*P* = 0.057, Fig. 7E), which is consistent with the resistant role identified in TCGA data. Moreover, according to half maximal inhibitory concentration (IC50) from Genomics of Drug Sensitivity in Cancer (GDSC), breast cancer cell lines with downregulation of ENSG00000230082 or ENSG00000247572 tended to be resistant to tamoxifen (Fig. S5).

## Discussion

Recent studies have demonstrated the regulatory role of lncRNAs and the utility of lncRNAs as potential diagnostic and prognostic biomarkers in breast cancer. However, lncRNA expression in individual breast cancer is notably heterogeneous, and only limited information regarding the IDElncRNA in an individual patient is available using population-level identification of differential expression. In this study, we used the *LncRIndiv* method to explore heterogeneous lncRNA expression in breast cancer and identify novel IDElncRNA-based TNBC subtypes. We demonstrated the high accuracy of the *LncRIndiv* method in paired breast cancer-normal tissue samples using cross-validation. In addition, FC distribution supported the reliability of IDElncRNAs, including IDElncRNAs that were consistently characterized by differential methylation and CNV at the individual level. *LncRIndiv* application for BRCA subtypes helps to identify subtype-specific IDElncRNAs and analyze their co-occurrence with genomic and epigenetic alterations. Moreover, some subtype-specific lncRNAs were drug response-related in clinical samples and cell lines, especially the luminal subtype-specific lncRNA ENSG00000230082 (*PRRT3-AS1*) for paclitaxel drug. Notably, TNBC subtype-specific prognostic IDElncRNAs could classify TNBC into two groups with distinct immunological characteristics. The patients with immune subtype had greater TMB, more infiltrating CD4 + T cells, and higher expression of immune checkpoint blocking genes to evade immune regulation than those in the patients with mesenchymal subtype. The high expression of immune signatures suggested that TNBC patients with immune subtype might potentially benefit from immune checkpoint inhibitors. Compared with the immune subtype, patients with the mesenchymal subtype mainly exhibited higher protein expression of EMT, a higher fraction of M2 macrophages, and fewer HRD as well as TMB.

Identification of IDElncRNAs is a fundamental step in the analysis of expression data. *LncRIndiv* identified some previously reported breast cancer-related lncRNAs in individuals. For example, oncogene *PVT1* was upregulated in 83 BRCA samples and suppressor *XIST* was downregulated in 180 BRCA samples [32–34]. As lncRNA expression might be regulated by aberrant promoter methylation and CNV, IDElncRNA is consistent with CNV or methylation alteration. However, some lncRNA dysregulation has no CNV or differential methylation in comparative analysis. There may be alternative regulatory mechanisms that affect lncRNA differential expression, such as miRNA expression [35]. Recent studies have revealed a comprehensive landscape of somatic mutations that affect the expression patterns of various genes, including lncRNAs [36]. However, it is still a challenge to assess the impact of mutations on lncRNA expression, which requires further detailed analysis.

As TNBC is more likely to respond to immunotherapy than other breast cancer subtypes, we found a group of TNBC patients who may respond to immune checkpoint inhibitors, especially TNBC patients with *PD-1* and *CTLA4* upregulation in the immune subtype. Some clinical studies have focused on evaluating the combination of CTLA4 and PD-1 blockers to support future research in combinatorial immunotherapy [37]. For the mesenchymal subtype, an increase in M2 tumor-associated macrophages was found to be correlated with primary tumor growth and metastatic spread [38]. Differential protein expression, such as mTOR, indicates the IL6-JAK-STAT3 signaling activation in the mesenchymal subtype. Thus, the mesenchymal subtype has the potential for treatment using a small-molecule inhibitor of mTOR.

LncRNA and mRNA expression were both altered in about 10% of BRCA samples. The low frequency of individual-level dysregulated expression suggests that cancer is highly heterogeneous in RNA expression. Moreover, module detection methods may help construct a multi-expression signature for subtypes with the same function module, which could be more efficient in clinical applications.

Further independent validation in the TNBC dataset should be undertaken to investigate the robustness of the classification in our future work. Currently, there are no public TNBC datasets with the expression of all prognostic lncRNAs and paired lncRNAs. In our study, we used TNBC cell lines to validate our conclusion. Although the TNBC cell line is the primary model for tumor cells, the lack of an immune microenvironment may distort the expression of immune system-related lncRNAs. With the continuous increase in bulk and single-cell sequencing data, the subtypes and differences in tumor immune infiltration can be further validated.

In summary, this study highlights the importance of IDElncRNAs in the characterization of inter-tumor heterogeneity in breast cancer. And the new TNBC subtypes indicate novel insights into TNBC immunotherapy. The *LncRIndiv* method can also be utilized in other cancers to comprehensively study IDElncRNAs. The statistical framework implemented in *LncRIndiv* enables the identification of differential expression without matched normal samples, which is more practical in clinical application. An important application of the individual lncRNA expression profile is to discover novel subtypes. Moreover, with the combination of genomic and epigenetic alteration information, *LncRIndiv* could be applied to construct patient-specific dysregulated networks for personalized medicine.

## Supplementary information

Supplemental Material

Table S2

Table S3

## Data Availability

All data generated or analyzed in this study are included in this published article and its Supplementary Information files.
