# Supplementary material for: Individualized lncRNA differential expression profile reveals heterogeneity of breast cancer
Source: Oncogene. 2021 Jun 15;40(27):4604–14. doi: 10.1038/s41388-021-01883-6 (PMC8266678; doi:10.1038/s41388-021-01883-6)
Supplement: Supplementary file 1 — Supplemental Material [file 41388_2021_1883_MOESM1_ESM.docx]

Individualized LncRNA Differential Expression Profile Reveals Heterogeneity of Breast Cancer

Zhangxiang Zhao^1#^, YingYing Guo^2,3#^, Yaoyao Liu^1#^, Lichun Sun^4^, Bo Chen^1^, Chengyu Wang^1^, Tingting Chen^1^, Yuquan Wang^1^, Yawei Li^1^, Qi Dong^1^, Liqiang Ai^1^, Ran Wang^5^, Yunyan Gu^1^*, Xia Li^6^*

^1^ Department of Systems Biology, College of Bioinformatics Science and Technology, Harbin Medical University, Harbin, China.

^2^ Department of Pharmacology (State-Province Key Laboratories of Biomedicine-Pharmaceutics of China, Key Laboratory of Cardiovascular Research, Ministry of Education), College of Pharmacy, Harbin Medical University, Harbin, China.

^3^ Northern Translational Medicine Research and Cooperation, Heilongjiang Academy of Medical Sciences, Harbin Medical University, Harbin, China.

^4^ Department of Breast Medical Oncology, Harbin Medical University Cancer Hospital, Harbin, China

^5^ Department of Physiology, Harbin Medical University, Harbin, China.

^6^ Department of Bioinformatics, College of Bioinformatics Science and Technology, Harbin Medical al University, Harbin, China.

# Contributed equally to this work.

***** Correspondence: Xia Li, College of Bioinformatics Science and Technology, Harbin Medical University, harbin, China. Email: lixia@hrbmu.edu.cn, Tel: +86-451-86615922; Yunyan Gu, Department of Systems Biology, College of Bioinformatics Science and Technology, Harbin Medical University, harbin, China. Email: guyunyan@ems.hrbmu.edu.cn, Tel: 86-451-8669-9584

**Contents**

[**Supplementary Methods** 3](#_Toc69737568)

[Data and preprocessing 3](#_Toc69737569)

[Identifying BRCA over-represented and subtype-specific lncRNAs 5](#_Toc69737570)

[Identifying prognosis-related lncRNAs and TNBC classification 6](#_Toc69737571)

[Pathway analysis of TNBC subtypes 6](#_Toc69737572)

[Characterization of the tumor immune microenvironment 6](#_Toc69737573)

[Cell culture and transfection 7](#_Toc69737574)

[Wound healing assay 7](#_Toc69737575)

[Transwell assay 8](#_Toc69737576)

[Immunofluorescence 8](#_Toc69737577)

[RNA extraction and quantitative real-time PCR 8](#_Toc69737578)

[Protein extraction and Western Blot 9](#_Toc69737579)

[Validation of TNBC subtype in CCLE 9](#_Toc69737580)

[Identifying BRCA drug response-related IDElncRNA 10](#_Toc69737581)

[Statistical 10](#_Toc69737582)

[**Supplementary tables** 12](#_Toc69737583)

[Table. S1. Statistics of samples and probes/genes of the TCGA BRCA multi-omics data 12](#_Toc69737584)

[**Supplementary figures** 13](#_Toc69737585)

[Fig. S1 13](#_Toc69737586)

[Fig. S2 14](#_Toc69737587)

[Fig. S3 16](#_Toc69737588)

[Fig. S4 17](#_Toc69737589)

[Fig. S5 18](#_Toc69737590)

[**Reference** 19](#_Toc69737591)

Supplementary Methods

## Data and preprocessing

Long non-coding RNA (lncRNA) expression profiles of breast cancer were obtained from the atlas of non-coding RNAs in cancer (TANRIC) characterized by reads per kilobase per million mapped reads [1]. Annotated information of lncRNAs in this dataset is archived in Gencode version 19.

LncRNA Individualization (*LncRIndiv*) (https://github.com/FuduanPeng/LncRIndiv) was used to identify differential expressed lncRNA in individual breast cancer patients based on pairwise comparison of 105 samples [2]. Stable lncRNA pairs were identified using a threshold of 95% normal samples.

RNA-SeqV2 mRNA expression profiles of 1080 breast cancer and 98 normal adjacent breast tissue samples from the Broad Institute GDAC Firehose (http://gdac.broadinstitute.org/runs/stddata__2016_01_28/data/BRCA/20160128/) were characterized by expectation maximization algorithm. Using the Rank Comparison (*RankComp*) algorithm in relative expression ordering analysis (REOA) software (https://github.com/pathint/reoa), we transformed continuous expression values of mRNAs to discrete values depending on the direction of differential expression [3]. The parameters of the REOA software were set as follows: sample type, 1; choice of algorithm, 1; job type, 2.

For somatic mutation data, we used Mutect2 somatic variant calls in mutation annotation format from the cancer genome atlas (TCGA) data portal (https://portal.gdc.cancer.gov). Somatic mutations annotated as silent mutations were removed.

Numeric focal-level copy number variation (CNV) values were generated using masked copy number segment files using GISTIC2 with a noise cutoff of 0.25. Peak regions were called using a threshold of q < 0.1 with the following parameters: cap values, 1.5; broad length cutoff, 0.7; confidence level, 0.99; joint segment size, 4; arm-level peel off, 1; maximum sample segments, 2000. The genes in the CNV region were screened by mRNA expression correlation (Spearman's rank-order correlation, *P <* 0.05; correlation coefficient > 0) [4].

DNA methylation profiles of 1083 cancer and 98 adjacent normal tissue samples from TCGA were used to extract individualized methylation profiles. CpG sites (25,978) measured by both the Illumina Human Methylation 27 Beadchip (27K) array and Illumina Human Methylation 450 Beadchip (450K) array were used in *RankComp*. The DNA methylation level of each probe was calculated by M/(U + M + 100), where *M* denotes the methylated signal intensity and *U* denotes the unmethylated signal intensity [5]. Probes with N/A values in any adjacent normal samples were removed, and 19,953 probes were annotated to genes according to the annotation table of the 27K platform. Based on the *RankComp* algorithm, probes’ methylation level was transformed to a discrete value depending on the hypomethylation or hypermethylation states. To investigate lncRNA genes’ DNA methylation, lncRNA probes were defined as probes located in the lncRNA promoter region. The mapping between lncRNAs and probes was obtained from Yang et al. [6]. For multiple probes mapping to the same lncRNA promoter, only probes with the same methylation alterations (hypermethylation or hypomethylation) were used to determine the methylation status of the lncRNA promoter.

Reverse phase protein array (RPPA) profiles of TCGA breast invasive carcinoma (BRCA) samples were collected from the Broad Institute GDAC Firehose, which contains 188 proteins from 937 samples.

Clinical information associated with TCGA breast invasive carcinoma (BRCA) samples, including the patient's overall survival time, clinical drug treatment records, measure of drug response, tumor stage, and age were obtained from TCGA data portal.

## Identifying BRCA over-represented and subtype-specific lncRNAs

The Prediction of microarray 50 (PAM50)-defined subtype of TCGA BRCA samples was obtained using the “TCGAbiolinks” R package [7]. HER2+ BRCA samples were divided into HER2+/HR+ and HER2+/HR− subtype (hormone receptor, HR). HER2- BRCA samples were divided into luminal A (HER2−/HR+) or luminal B (HER2−/ER+/PR−) subtypes (estrogen receptor, ER; progesterone receptor, PR). The triple-negative breast cancer (TNBC) subtype is defined as ER-, PR-, and HER2- tumor. We performed a hypergeometric test to identify over-represented individual differentially expressed lncRNAs (IDElncRNAs) for each subtype (*P* < 0.05). The lncRNAs that are differentially expressed in only specific BRCA subtype are defined as subtype-specific IDElncRNAs. For each subtype-specific lncRNAs, co-occurrences with mutation, CNV, and differential methylation were calculated using the hypergeometric test in each subtype.

## Identifying prognosis-related lncRNAs and TNBC classification

Univariate Cox proportional hazards regression model was used to test the association between IDElncRNAs and survival time of TNBC patients. Cox regression coefficients and corresponding *P*-values were determined for IDElncRNAs with differential expression frequencies of 5%–95%. We used hierarchical clustering analysis (Ward.D2 method) to identify subtypes in TNBC.

## Pathway analysis of TNBC subtypes

We performed a hypergeometric test to identify subtype-specific protein-coding genes. A protein-coding gene was defined as subtype-specific if the frequency of the gene was significantly enriched in only one TNBC subtype (*P* < 0.01). Kyoto encyclopedia of genes and genomes (KEGG) pathway enrichment analysis was performed using the “KEGGProfile” R package (https://github.com/slzhao/KEGGprofile). Pathways with *P* < 0.05 and pathway size > 20 were considered as significantly enriched pathways. Protein expression was compared between the two subtypes using Wilcoxon rank-sum test.

## Characterization of the tumor immune microenvironment

Tumor mutational burden (TMB) was defined as the mutation count in a sample, whereas non-silent TMB was defined as the non-silent somatic mutation count. Comparisons between TNBC subtypes in TMB and homologous recombination deficiency (HRD) scores were performed using the Wilcoxon rank-sum test.

Wilcoxon rank-sum test was used to compare immune cell compositions. LncRNA-pathway pairs with significant lncRES scores (*P* < 0.05) in BRCA were obtained. A hypergeometric test was performed to identify co-expressed lncRNAs and immune genes in TNBC*. P*-values were adjusted using the Benjamini-Hochberg procedure. Significantly co-expressed lncRNA-immune gene pairs were used to construct the immune regulatory network (adjusted *P*-values < 0.1).

## Cell culture and transfection

Human triple-negative breast cancer cell line MDA-MB-231 was purchased from the Cell Bank of the Chinese Academy of Sciences (Shanghai, China). Cells were cultured in medium (Leibovitz L-15 Medium; Biological Industries, Israel) containing 10% fetal bovine serum (Sigma-Aldrich, USA), 100 U/mL penicillin, and 100 U/mL of streptomycin at 37 °C and 75% humidity in a 100% air incubator. The overexpression plasmid of lncRNA *PTOV1-AS1* was constructed by pcDNA3.1. siRNA against lncRNA *PTOV1-AS1* (si-PTOV1-AS1) was constructed by RiboBio Tech (Guangzhou, China). Lipofectamine2000 (Invitrogen, CA) was used for transfection according to the manufacturer’s instructions. 10 ng/mL TGF-β1 (Sigma-Aldrich, USA) was added into the medium and cells were cultured for 48 h.

## Wound healing assay

Cells were seeded in 6-well plates until they formed a monolayer fusion. A 100 µL pipette tip was used to form a wound after transfection. PBS was used to wash the non-adherent cells. The wounded areas were observed and photographed under a microscope (×20 objective) at 0, 24, 48 h after the scratch. The scratched images were analyzed by using Image J.

## Transwell assay

24-well plates with Transwell membrane (8.0 µm, Corning, USA) were used for migration (without Matrigel) and invasion assays (with Matrigel). 5×104 cells in serum-free medium were seeded into the upper chamber. The bottom well was filled with Leibovitz L-15 Medium containing 10% fetal bovine of serum to stimulate migration and invasion. Following 48 h of incubation at 37 °C, the cells were fixed with 4% paraformaldehyde and stained with 0.1% crystal violet. Migrating cells were counted by Image J.

## Immunofluorescence

Cells were seeded in 24-well plates and transfected or treated with TGF-β1 for 48 h. Cells were washed three times with PBS and fixed with 4% paraformaldehyde for 30 min. Then added penetrating solution (4 μL Triton X-100 + 0.1 g BSA + 1 mL PBS) for 5 min and blocked with 50% goat serum at 37 °C for 30 min. Primary antibody against ZO-1 (1:200, 21733-1-AP, Proteintech, Rosemont, IL, USA) was incubated overnight at 4 °C. The next day, they were incubated with anti-rabbit IgG (H+L) (Alexa Fluor 488 Conjugate; 1:500, 4412, CST, Danvers, USA) at 25 °C. DAPI staining nucleus for 5 min. Images were photographed under a fluorescence microscope.

## RNA extraction and quantitative real-time PCR

The total RNA of cell samples was extracted by TRIzol reagent. The concentration of RNA was determined by NanoDrop8000 (Thermo, USA). RNA reverse transcribed for cDNA by using 5× All-in-One RT Master Mix (TransGen Biotech, China). All cDNA samples were prepared into a 20 μL reaction system. GAPDH was used as the internal control. The relative mRNA level was calculated based on the cycle threshold (Ct) values.

## Protein extraction and Western Blot

The total protein was extracted from the cells with RIPA lysis buffer containing protease inhibitor (Beyotime, China). The protein samples (50 μg) were separated with 10% SDS-PAGE gel electrophoresis and transferred to a pure nitrocellulose membrane. The primary antibodies anti-ZO-1 (1:500, 21733-1-AP, Proteintech, Rosemont, IL, USA), anti-Vimentin (1:500, 10366-1-AP, Proteintech, Rosemont, IL, USA), anti-GAPDH (1:500, 60004-1-Ig, Proteintech, Rosemont, IL, USA) and anti-E-Cadherin (1:500, ab76055, Abcam, Cambridge, UK) were incubated overnight at 4 °C. The protein bands were developed and analyzed by Odyssey infrared imaging system (Odyssey CLX, Biosciences, USA).

## Validation of TNBC subtype in CCLE

Based on the same clustering method and lncRNAs in TCGA BRCA samples, the IDElncRNA expression profile of TNBC cell lines was classified into two classes (class 1 and class 2). Protein expression was compared between the two classes using Wilcoxon rank-sum test, and drug responses were compared using the Welch’s t-test.

## Identifying BRCA drug response-related IDElncRNA

In TCGA, BRCA patients with “complete response” were considered as the sensitive group; BRCA patients with “stable disease”, “clinical progressive disease”, and “partial response” were considered as the resistant group. We selected the top 10 most used drugs that were administered to both sensitive and resistant BRCA patients (n > 3) for further analysis. Fisher’s exact test was used to analyze the independence between differential expression of IDElncRNA and drug response in all BRCA patients. IDElncRNAs with *P*-values < 0.05 were defined as drug response-related IDElncRNAs.

For drug response validation in cell lines, lncRNA expression profile and ActArea values of 51 breast cancer cell lines were obtained from Broad Institute cancer cell line encyclopedia (CCLE) (https://data.broadinstitute.org/ccle) and individualized using *LncRIndiv* [8]. Breast cancer cell lines were divided into two groups according to differential expression of lncRNA. Two-tailed Welch’s t-test was used for ActArea value comparisons after the normal distribution test. Tamoxifen-resistant lncRNAs were evaluated based on the drug response data from Genomics of Drug Sensitivity in Cancer (GDSC2, https://www.cancerrxgene.org/downloads/) and expression data from Cell Model Passports (https://cellmodelpassports.sanger.ac.uk/downloads) [9, 10]. Breast cancer cell lines were divided into two groups according to each lncRNA’s differential expression. Welch’s t-test was performed to compare half maximal inhibitory concentration (IC50) between groups.

Statistical Analysis

Bioinformatics analysis, statistical and hierarchical clustering analyses were performed using R software version 3.6.1*.* Normality tests were performed using the Shapiro-Wilk normality test. The homogeneity of variance test was performed by Levene test for each gene. If the *P*-value > 0.05, data were considered normally distributed and satisfied homogeneity of variance. In this study, most genes were normally distributed and satisfied homogeneity of variance. For experiment analysis, data were presented as mean ± SEM. One-way analysis of variance (ANOVA) was used for multiple comparisons. Bonferroni test was used for correction and a two-tailed *P* < 0.05 was considered statistically significant. Statistical analyses were carried out using GraphPad Prism 8.0.

# Supplementary tables

## Table. S1. Statistics of samples and probes/genes of the TCGA BRCA multi-omics data

| **Data Type** | **Platform** | **Number of Tumor Samples** | **Number of Normal Samples** | **Number of Probes/Genes** |
| --- | --- | --- | --- | --- |
| mRNA expression | Illumina HiSeq | 1080 | 98 | 16033 |
| DNA methylation | Illumina HumanMethylation27/  Illumina HumanMethylation450 | 1083 | 98 | 19953 |
| Copy number variation | Affymetrix SNP 6.0 | 1111 | / | 27100 |
| Somatic mutation | Illumina HiSeq 2000 | 986 | / | 18847 |
| LncRNA expression | Illumina GA | 837 | 105 | 12727 |

# Supplementary figures

## Fig. S1


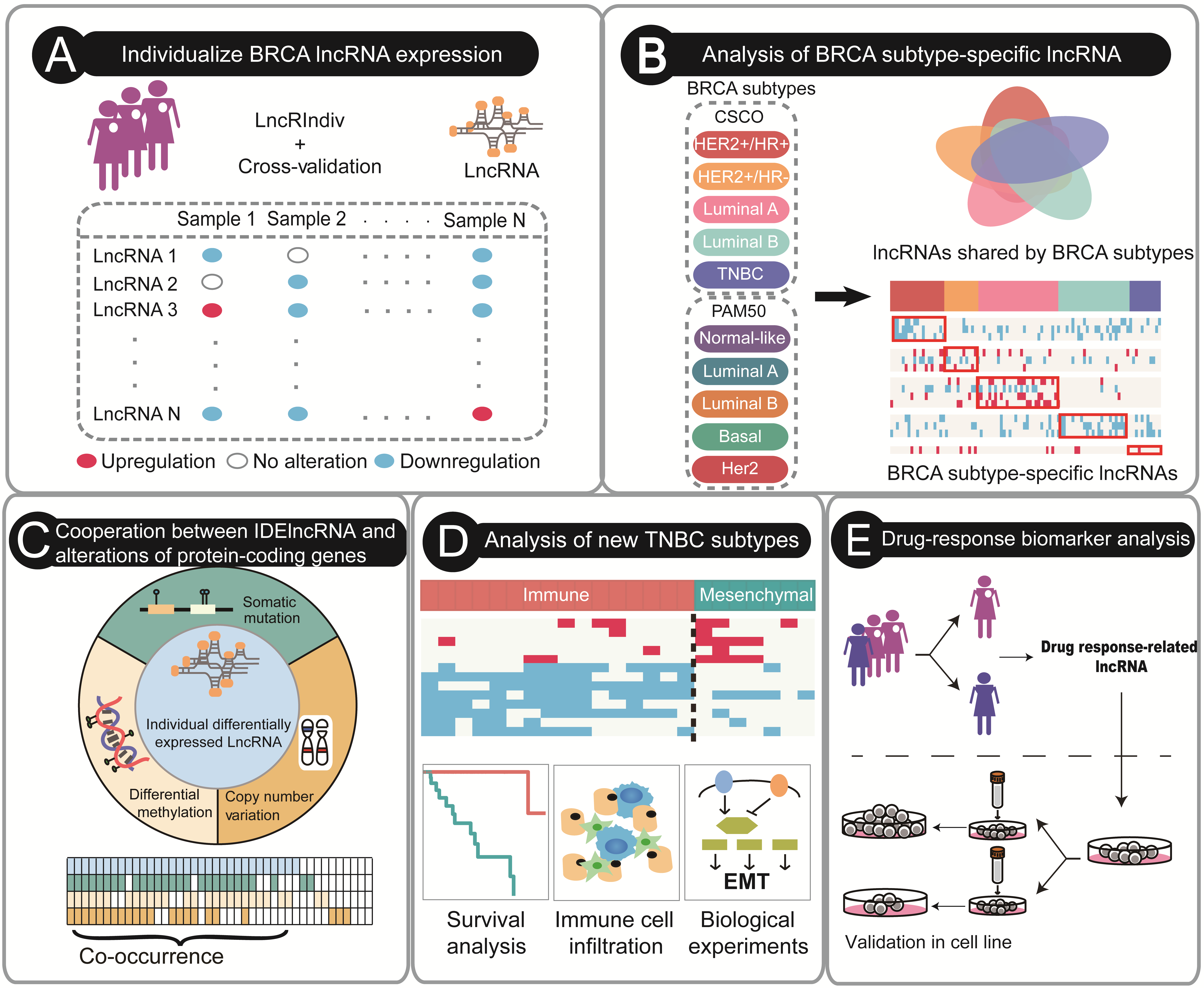


**Fig. S1. Flowchart of the study.**

## Fig. S2





**Fig. S2. BRCA PAM50 subtype-specific lncRNAs.**(A) The upset diagram shows the subtype-specific lncRNAs and overlappings of over-represented lncRNAs among breast cancer subtypes. The black horizontal bar graphs indicate the number of over-represented lncRNAs of each subtype. Black circles show the overlappings between two subtypes and the black vertical bar graphs show the number of overlapped subtype over-represented lncRNAs. Colored bars represent the amount of subtype-specific lncRNAs.

(B) Heatmap of breast cancer PAM50 subtype-specific lncRNAs. The row and column represent lncRNAs and samples, respectively. Black rectangular indicates each subtype of breast cancer and corresponding subtype-specific lncRNAs. The right bar displays the proportion of BRCA samples in which the subtype-specific lncRNAs are differentially expressed. For clarity, we presented the lncRNAs that are differentially expressed in more than 5% samples within each subtype.

## Fig. S3


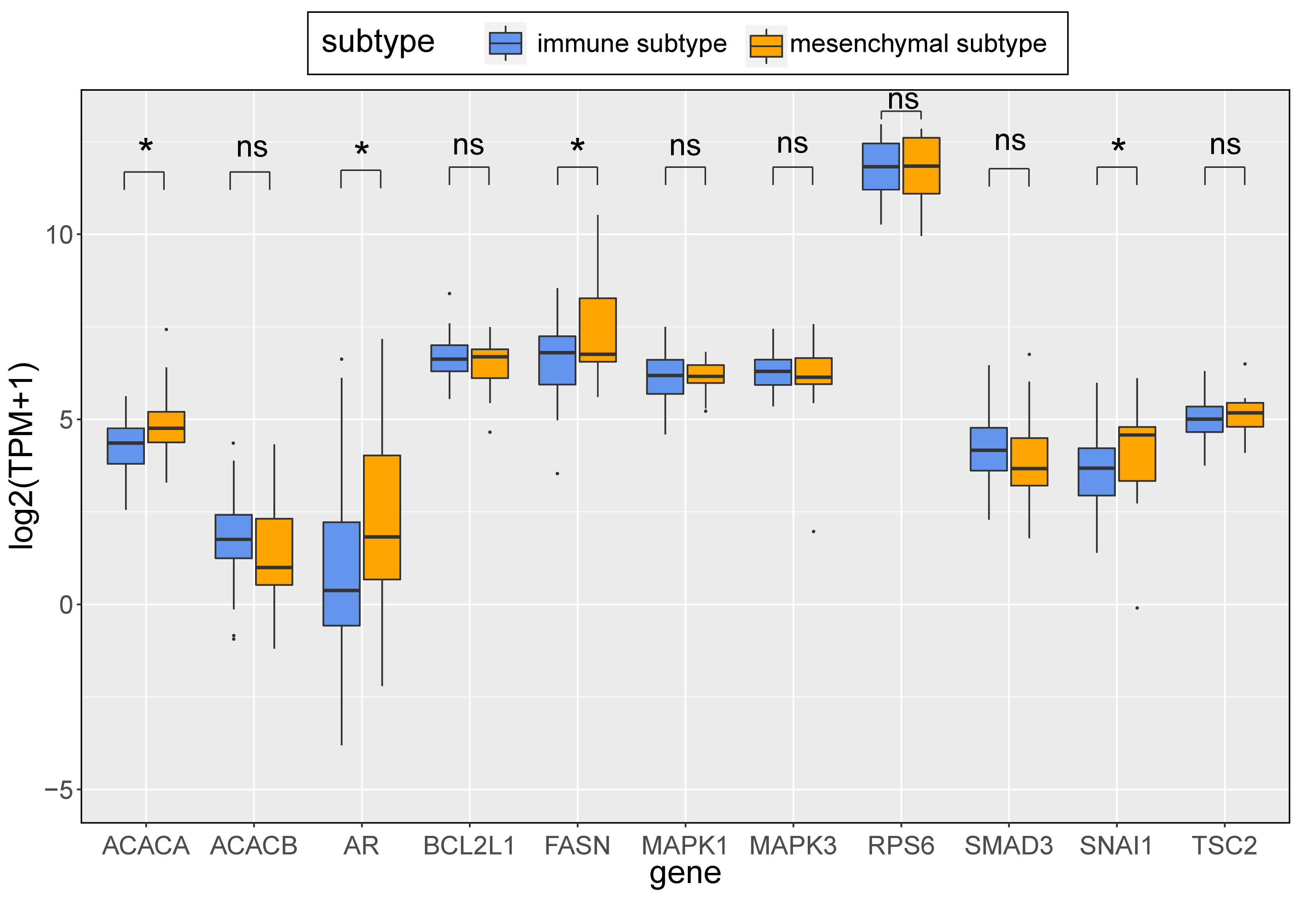


**Fig. S3.** **Differentially expressed mRNAs between TNBC mesenchymal subtype and immune subtype.** Blue and yellow boxes represent mRNA expression (scaled using log2) in TNBC immune subtype and mesenchymal subtype, respectively. The data were analyzed using the Wilcoxon rank-sum test. Statistical significance is indicated by * *P* ˂ 0.05 and non-significant difference (ns).

## Fig. S4


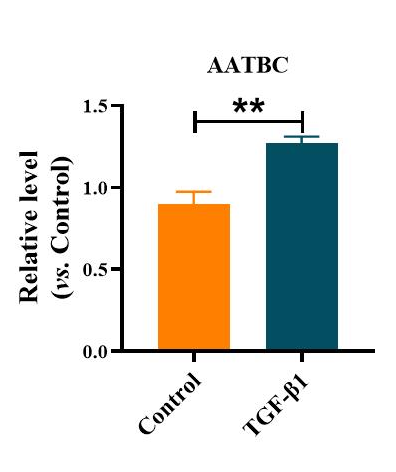


**Fig. S4.** **mRNA expression of lncRNA *AATBC* in** **MDA-MB-231 cells treated with TGF-β1.** qRT-PCR analysis of the expression of lncRNA AATBC in MDA-MB-231 cells. n=4. ** *P* < 0.01.

## Fig. S5


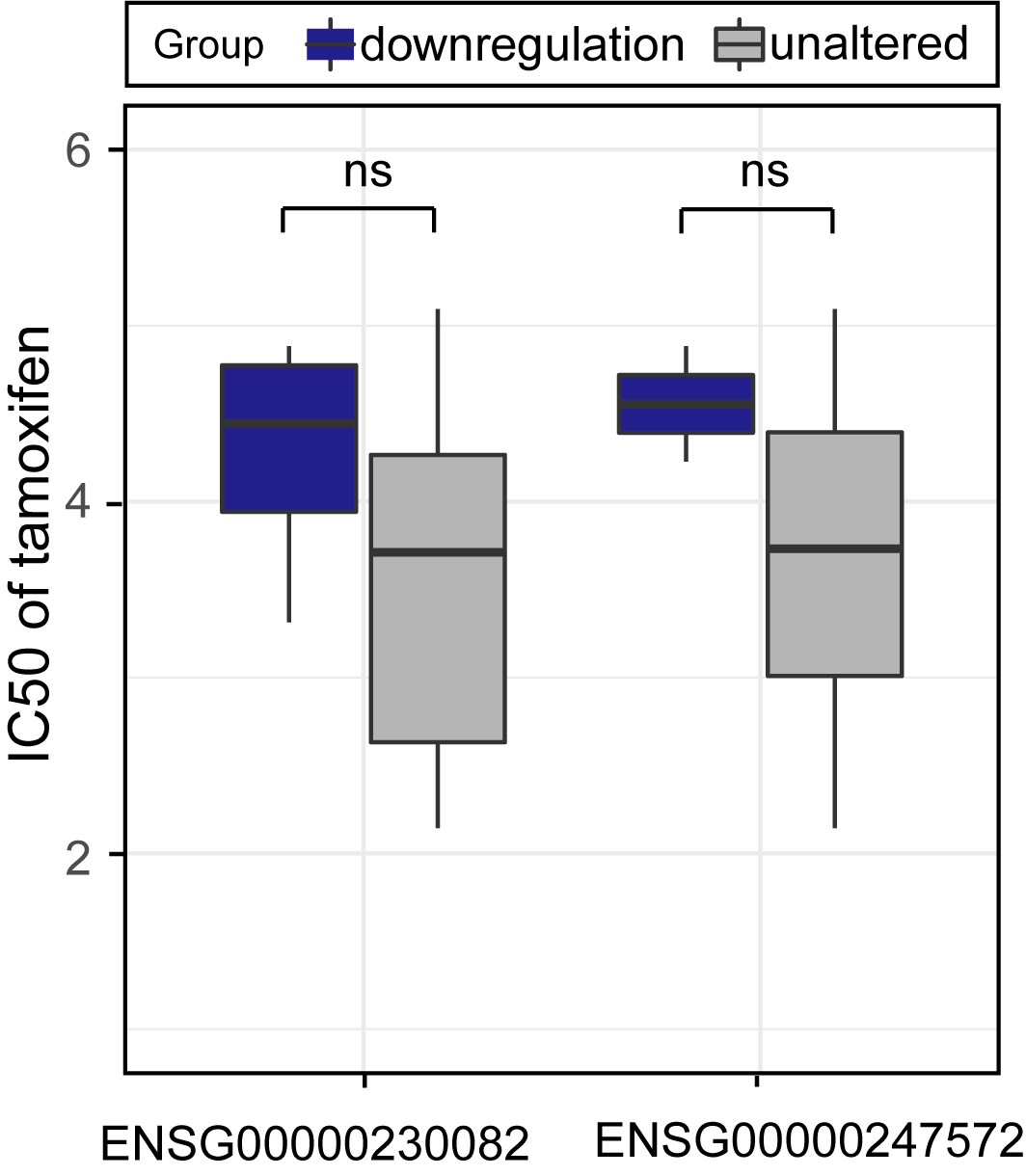


**Fig. S5. IC50 values of tamoxifen between breast cancer cell lines in the GDSC2 database.** Blue boxes represent cell lines with tamoxifen response-resistant lncRNA differential expression and gray boxes represent cell lines with unaltered tamoxifen response-resistant lncRNA. Statistical significance is indicated by non-significant difference (ns).

Reference

1. Li J, Han L, Roebuck P, Diao L, Liu L, Yuan Y *et al*. TANRIC: An Interactive Open Platform to Explore the Function of lncRNAs in Cancer. Cancer Res. 2015; 75: 3728-3737.

2. Peng F, Wang R, Zhang Y, Zhao Z, Zhou W, Chang Z *et al*. Differential expression analysis at the individual level reveals a lncRNA prognostic signature for lung adenocarcinoma. Mol Cancer. 2017; 16: 98.

3. Wang H, Sun Q, Zhao W, Qi L, Gu Y, Li P *et al*. Individual-level analysis of differential expression of genes and pathways for personalized medicine. Bioinformatics. 2015; 31: 62-68.

4. Zhou W, Zhao Z, Wang R, Han Y, Wang C, Yang F *et al*. Identification of driver copy number alterations in diverse cancer types and application in drug repositioning. Mol Oncol. 2017; 11: 1459-1474.

5. Dedeurwaerder S, Defrance M, Calonne E, Denis H, Sotiriou C, Fuks F. Evaluation of the Infinium Methylation 450K technology. Epigenomics. 2011; 3: 771-784.

6. Wang Z, Yang B, Zhang M, Guo W, Wu Z, Wang Y *et al*. lncRNA Epigenetic Landscape Analysis Identifies EPIC1 as an Oncogenic lncRNA that Interacts with MYC and Promotes Cell-Cycle Progression in Cancer. Cancer cell. 2018; 33: 706-720 e709.

7. Colaprico A, Silva TC, Olsen C, Garofano L, Cava C, Garolini D *et al*. TCGAbiolinks: an R/Bioconductor package for integrative analysis of TCGA data. Nucleic Acids Res. 2016; 44: e71.

8. Ghandi M, Huang FW, Jane-Valbuena J, Kryukov GV, Lo CC, McDonald ER, 3rd *et al*. Next-generation characterization of the Cancer Cell Line Encyclopedia. Nature 2019; 569: 503-508.

9. Yang W, Soares J, Greninger P, Edelman EJ, Lightfoot H, Forbes S *et al*. Genomics of Drug Sensitivity in Cancer (GDSC): a resource for therapeutic biomarker discovery in cancer cells. Nucleic Acids Res. 2013; 41: D955-961.

10. van der Meer D, Barthorpe S, Yang W, Lightfoot H, Hall C, Gilbert J *et al*. Cell Model Passports-a hub for clinical, genetic and functional datasets of preclinical cancer models. Nucleic Acids Res. 2019; 47: D923-D929.
